# 10‐year follow‐up in the assessment on P300 in older adults with mild cognitive impairment: a case series

**DOI:** 10.1002/alz70860_103926

**Published:** 2025-12-23

**Authors:** Juliana de Sá Novaes, Michele da Rocha Anselmo, Gabriela Tomé Oliveira Engelmann, Bruna Fulgêncio Dias, Bernardo de Mattos Viana, Maria Aparecida Camargos Bicalho, Ludimila Labanca

**Affiliations:** ^1^ Universidade Federal de Minas Gerais, Belo Horizonte, Minas Gerais, Brazil; ^2^ Cog‐Aging Research Group, Belo Horizonte, Minas Gerais, Brazil; ^3^ Cog‐Aging Research Group, Universidade Federal de Minas Gerais (UFMG), Belo Horizonte, Minas Gerais, Brazil; ^4^ Universidade Federal de Minas Gerais, Belo Horizonte, Brazil; ^5^ Sciences Applied to Adult Health Postgraduate Program, School of Medicine, Universidade Federal de Minas Gerais (UFMG), Belo Horizonte, Minas Gerais, Brazil; ^6^ Federal University of Minas Gerais, Belo Horizonte, Minas Gerais, Brazil; ^7^ Department of Clinical Medicine, Faculty of Medicine, Universidade Federal de Minas Gerais (UFMG), Belo Horizonte, Minas Gerais, Brazil; ^8^ Geriatrics and Gerontology Center Clinical Hospital of Universidade Federal de Minas Gerais, Belo Horizonte, Minas Gerais, Brazil; ^9^ National Institute of Science and Technology Neurotec R (INCT‐MM), Belo Horizonte, Minas Gerais, Brazil; ^10^ Older Adult's Psychiatry and Psychology Extension Program (PROEPSI), School of Medicine, Universidade Federal de Minas Gerais (UFMG), Belo Horizonte, Minas Gerais, Brazil

## Abstract

**Background:**

In Brazil, the older adult population has already surpassed 32 million people (IBGE, 2023). Cognitive decline stands as one of the most important chronic‐degenerative disabling changes, which can generate Major or Mild Neurocognitive Disorders (Bárrios 2020; American Psychiatric Association, 2023). The third most common disease in the older adult population is presbycusis (hearing loss associated with aging) (Homans, 2016) and there is increasing evidence of the relationship between reduced hearing acuity and functional decline, depression, social isolation and cognitive decline and dementias (Livingston, 2020). In this context, it is needed to carry out and improve studies with the elderly population, especially to monitor and describe the aging process, ensuring a greater quality of life during old age.

**Method:**

Six older adults were evaluated in 2013 and 2023. They underwent medical and neuropsychological evaluation, audiological anamnesis, audiological evaluation and the P300 electrophysiological exam.

**Result:**

Of the six cases presented here, 2 patients are male and 4 are female. The average age in 2013 was 73.5 years, with a minimum age of 66 and a maximum of 80. In 2023, the average age was 83.5 years. Three showed improvement in their cognitive status, one remained stable and two showed worsening. Recent studies have proven that Mild Cognitive Impairment cases are able to show general improvement or even go into remission (Souza, 2019), which has happened in four out of the six cases. The cases with identified remission of cognitive decline were the only one in which educational years were over 10 (stable case) and one with hearing loss treatment (went into remission). Studying years stimulus can delay cognitive decline in older age (Feldberg, 2021) and the use of hearing aids can also be a protection (Cantuaria, 2024). It was found that P300 latency was increased (latency exceeding 450ms) (Raggi, 2013) in the two cases classified as dementia.

**Conclusion:**

The study suggests that the result of the non‐invasive electrophysiological test P300 can be a tool to help in the dementia diagnosis.

